# Urologic malignancy risk with chronic tumor necrosis factor-alpha inhibitor (TNF-I) exposure: a multicenter, retrospective cohort study

**DOI:** 10.1007/s00345-026-06309-0

**Published:** 2026-02-26

**Authors:** Conor B. Driscoll, Jordan M. Rich, Christopher Yang, Joseph Nicolas, Dylan Isaacson, Philip Silberman, Xinlei Mi, Sai Kaushik Shankar Ramesh Kumar, Hui Zhang, Steven Belknap, William H. Temps, Edward M. Schaeffer, Shilajit D. Kundu

**Affiliations:** 1 Department of Urology, Northwestern University Feinberg School of Medicine, 676 N. St. Clair Street, Arkes 23, Chicago, IL 60611 USA; 2 Department of Urology, New York University, New York, NY USA; 3 Department of Dermatology, Northwestern University Feinberg School of Medicine, Chicago, IL USA; 4 Department of Urology, Cleveland Clinic Foundation, Cleveland, OH USA; 5 Department of Information Technology, Northwestern University Feinberg School of Medicine, Chicago, IL USA; 6 Division of Biostatistics, Department of Preventative Medicine, Northwestern University Feinberg School of Medicine, Chicago, IL USA

**Keywords:** Urologic cancer, Prostate cancer, Tumor necrosis factor alpha inhibitors, Inflammation

## Abstract

**Purpose:**

Chronic inflammation has been linked to oncogenesis, including in prostate cancer (PCa). Tumor Necrosis Factor (TNF) has been implicated in many of these chronic inflammatory pathways. We assessed urologic malignancy risk in patients with long-term exposure to TNF inhibitors (TNF-I).

**Methods:**

Retrospective non-matched cohort study of patients with chronic inflammatory conditions from 1996 to 2023. TNF-I exposure was identified using medications. The unmatched control cohort consisted of TNF-I-unexposed patients with the same chronic inflammatory conditions. Urologic malignancies identified using ICD-10 codes and manual chart review. Inverse Probability of Treatment Weighting was used to balance distributions across exposure groups. Hazard ratios (HR) were estimated using multivariable regression followed by logistic regression for relative risk (RR) on sub-analysis.

**Results:**

There were 13,377 patients with TNF-I exposure and 42,832 patients without TNF-I exposure. TNF-I exposure was negatively associated with PCa (HR 0.50, 95% CI 0.28–0.90, *P* = 0.02), but with higher Gleason grade group (RR 1.11, 95% CI 1.01–1.22, *P* = 0.04). TNF-I exposure was not associated with bladder cancer diagnosis (HR 0.71, 95% CI 0.26–1.95, *P* = 0.51) but had increased risk of multifocal tumor development (*P* = 0.001) and high-grade tumor (*P* = 0.004). TNF-I exposure was not associated with kidney cancer risk (HR 1.47, 95% CI 0.85–2.54, *P* = 0.17) but with increased risk of higher clinical stage (RR 2.01, 95% CI 1.21–3.33, *P* = 0.01).

**Conclusion:**

TNF-I exposure was associated with lower risk of PCa but higher-grade group PCa. TNF-I exposure was associated with higher risk of multifocal and high-grade bladder cancer and of higher stage kidney cancer. TNF-I exposed patients may need modified urologic cancer screening.

**Patient summary:**

In this report, we looked at exposure to TNF inhibitor medications and their risk of prostate, bladder, and kidney cancer. We found that, compared to patients who did not take these medications, there was a lower risk of prostate cancer but with higher grade disease. There was no change in the overall risk of bladder or kidney cancer diagnosis, but they did have worse features. Further studies of patients on TNF inhibitors should be performed to confirm these findings.

**Supplementary Information:**

The online version contains supplementary material available at 10.1007/s00345-026-06309-0.

## Introduction

Recent data suggests higher incidence of clinically significant prostate cancer (PCa) in men with inflammatory bowel disease (IBD), with Burns et al. demonstrating a fourfold increased risk of high-grade PCa in these patients [1]. Previous epidemiologic studies note an association between chronic inflammation and PCa along with other malignancies, including colon and skin cancer, in patients with IBD [2–4]. The risk of urologic tumors in patients with chronic inflammatory conditions, including but not limited to IBD, is not well elucidated [5, 6].

Key drivers of malignant transformation include longstanding inflammation, chronic immunosuppression, and shared genetic pathways. A common immunosuppressive medication class in patients with chronic inflammatory disorders is the Tumor Necrosis Alpha Inhibitors (TNF-I), with 5 approved medications (Adalimumab, Infliximab, Etanercept, Golimumab, Certolizumab) with $42 billion in market cap in 2023 [7]. Tumor necrosis factor-alpha (TNF) is a widely expressed cytokine with varying systemic effects. TNF is a homotrimer protein of 157 amino acids, mainly generated by activated macrophages, T-lymphocytes, and natural killer cells. It exists in both soluble and transmembrane forms and triggers a large inflammatory cascade. TNF can be tumoricidal and induce apoptosis and cell death via binding to the M1 death domain or promote tumor growth by binding to the M2 proliferation domain [8].

The established literature cites TNF-I as low risk for malignancy development, aside from Non-Hodgkin’s Lymphoma (NHL) and non-melanoma skin cancer (NMSC), however, there is a signal that TNF-I exposure may decrease the risk of PCa [9]. PCa development has been shown to be influenced by chronic inflammation, providing a feasible pathway for this protective effect of TNF-I [10]. Similarly, renal cell carcinoma (RCC) is susceptible to immune regulation, as immune checkpoint inhibitors are first line treatments in advanced disease [11]. This relationship warrants further investigation as there are no studies that specifically evaluate urologic cancer risk after TNF-I exposure [12]. Here we investigate urologic malignancy risk in patients on long-term TNF-I immunosuppression through a multi-hospital retrospective cohort.

## Materials and methods

### Study design

Observational retrospective cohort study.

### Data extraction

Institutional Review Board approval was granted by Northwestern University (IRB# STU00211830) prior to data collection and analysis. The Northwestern Medicine Enterprise Data Warehouse, comprising 11 hospitals in 8 counties in and around Chicago, IL [13], was queried for adults (≥ 18 years) who presented between July 1996 and July 2023 with a chronic inflammatory condition for which TNF-I is indicated.

Patients exposed to TNF-I were identified using generic medication names and chronic inflammatory condition for which they were prescribed. Chronic inflammatory conditions were grouped based on on-label indications in Supplementary Methods 1. Urologic malignancies [PCa, urothelial cell carcinoma (UCC), RCC] occurring after TNF-I initiation were identified using ICD-9/10 codes (Supplementary Methods 2) [14]. Demographic data, comorbidities and dates of physician encounters were extracted. Overall follow-up time was defined as duration between first documented and most recent documented physician encounter. Post-exposure follow-up time for TNF-I exposed patients was defined as duration between first TNF-I prescription and most recent documented physician encounter [15].

### Control groups

Patients with chronic inflammatory diseases but without TNF-I exposure were controls.

### Covariates

Medical comorbidities were queried using ICD-9/10 codes. For PCa, additional data was extracted using ICD-10-CM and CPT codes and included in multivariable analyses (Supplementary Methods 3). Among patients with TNF-I and malignancy, TNF-I-related covariates, including index disease, dose, and disease duration, were determined by chart review. PSA tests after PCa diagnosis were excluded.

### Outcomes

The primary outcome was urologic malignancy diagnosis stratified by TNF-I exposure and, if exposed, type of TNF-I medication. Tumor stage at diagnosis, topography, and histologic subtype were determined from biopsy or surgical pathology if available. Metastatic disease (N + or M+) and stage at diagnosis was assessed via chart review.

The secondary PCa outcomes were: (1) Clinical TNM stage at diagnosis, comparing T1 to ≥T2; (2) Pathologic TNM staging on prostatectomy, comparing ≤T2 to ≥T3; (3) Gleason score, comparing low/intermediate-risk (≤3 + 4) to higher-risk (≥4 + 3) disease. The secondary RCC outcomes were: (1) Clinical TNM stage at diagnosis; (2) Pathologic TNM Stage at diagnosis; (3) partial nephrectomy; (4) radical nephrectomy. The secondary UCC outcomes were: (1) Pathologic TNM stage at diagnosis, comparing Stage 1 to Stage ≥2; (2) Pathologic Grade; (3) Number of locations; (4) radical cystectomy.

### Statistical analysis

Subjects were stratified based on prior exposure or non-exposure to TNF-I. Demographic and clinical variables were presented as medians and interquartile ranges (IQR) for continuous variables and compared using Wilcoxon rank sum test. Categorical variables were summarized as counts and percentages and compared using Fisher’s exact and Chi-squared. Any patient with a non-NMSC malignancy prior to TNF-I exposure was excluded. We compared cancer rates between TNF-I exposed and unexposed cohorts using Cox proportional hazards regression analysis, adjusting for age, race, sex, smoking status and underlying inflammatory disease. PSA was analyzed as a continuous variable. The proportional assumption was checked prior to model fitting. TNF-I exposure was considered a time-varying factor, as TNF-I induction date was not fixed in the exposed group [16]. Models were fit incorporating patients who developed any malignancy during the study period then individually for each cancer type. In all models, TNF-I exposed and unexposed patients who did not develop malignancies were included. For PCa, we included only male subjects. A multivariable Cox regression sensitivity analysis for PCa was performed with number of screening PSA’s as an additional covariable.

Logistic regression was used to assess relative risk (RR) of malignancy based on TNF-I exposure. Propensity scores were estimated using a multivariable logistic regression model with age, race, gender, and smoking status as predictors of exposure. Inverse probability weights were calculated as 1/propensity scores for TNF-I exposed patients and as 1/(1-propensity score) for unexposed patients. Inverse Probability of Treatment Weighting was used to balance the covariate distributions in the logistic regression model for RR of malignancy [17–21]. Analysis was performed both for Gleason score ≤ 7 vs. ≥8 and Grade Group (GG) ≤ 2 vs. ≥3. Regression models were presented as forest plots with hazard ratios (HR) and 95% confidence intervals (CI) for the Cox regression model, and as RR with 95% CI for the logistic regression model. *P* < 0.05 was considered statistically significant in a two-sided manner. Statistical analysis was performed using R (4.2.0).

## Results

Of 56,209 patients with chronic inflammatory disorders, 13,337 had TNF-I exposure (23.7%). For the PCa analysis, there were 19,678 men of whom 5,154 (26.2%) were TNF-I exposed.

**Fig. 1:**
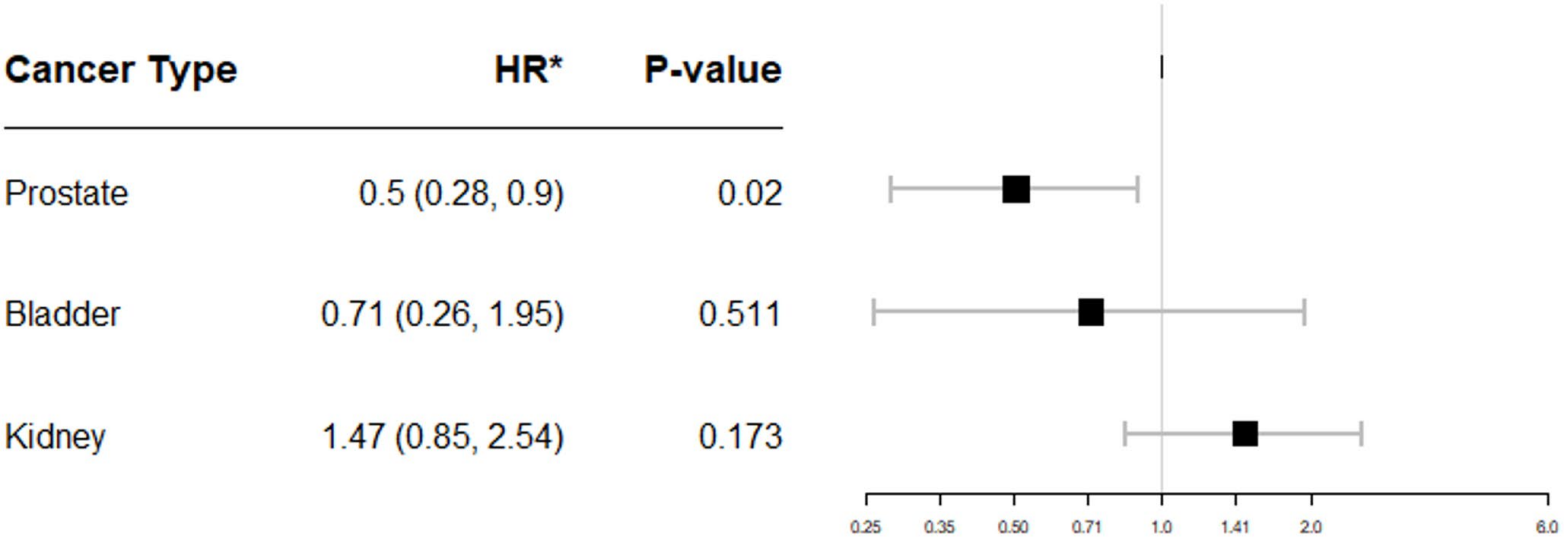
Forest plot of odds ratio for urologic cancers for TNF-I exposed patients compared to TNF-I unexposed patients

Baseline demographics for each cohort are listed in Supplementary Tables 1–3. There was a significantly decreased risk of PCa (HR 0.50, 95% CI 0.28–0.90, *P* = 0.02) in TNF-I exposed patients (Fig. 1). There was no significant change in PCa risk by inflammatory disease type (Supplementary Fig. 1). Using propensity matching for the secondary PCa outcomes, there was an increased number of elevated PSA’s prior to diagnosis (RR 1.23, 95% CI 1.08–1.39, *P* = 0.001) and higher GG at diagnosis (RR 1.11, 95% CI 1.01–1.21, *P* = 0.036) in TNF-I exposed patients (Table 1). When stratified by individual drug, analysis re-demonstrated increased risk of higher GG PCa in patients exposed to etanercept but a decreased risk in patients exposed to either adalimumab or infliximab (Supplementary Tables 4–6). Multivariable sensitivity analysis controlling for number of screening PSAs found PCa diagnosis risk remained significantly decreased (HR 0.50, 95% CI 0.27–0.91, *P* = 0.023) (Supplementary Fig. 2).

For patients with RCC, there was no significant change in diagnosis risk (HR 1.47, 95% CI 0.85–2.54, *P* = 0.173) (Fig. 1) nor baseline differences in surgical approach, histologic subtype, recurrence, or median year of diagnosis, but there was a significant difference in mortality, with no deaths in TNF-I exposed patients (*P* = 0.015) (Supplementary Table 2). For RCC secondary outcomes, there was a significantly increased risk of higher clinical stage (RR 2.01, 95%CI 1.21–3.33, *P* = 0.013) at diagnosis in TNF-I exposed patients (Table 1). Patients exposed to adalimumab had higher risk of partial nephrectomy and lower risk of radical nephrectomy while those exposed to infliximab re-demonstrated an increased risk of higher clinical stage at diagnosis (Supplementary Tables 4–6).

For the patients with UCC, there was no significant change in diagnosis risk (HR 0.71, 95% CI 0.26–1.95, *P* = 0.511) (Fig. 1) nor in baseline characteristics (Supplementary Table 3). For secondary UCC outcomes in TNF-I-exposed patients, there was a significantly increased risk of multifocal tumor development (*P* = 0.001) and high-grade tumor (*P* = 0.004) but lower risk of higher pathological stage at diagnosis (*P* = 0.007) and radical cystectomy (*P* = 0.003) (Table 1). In patients exposed to Adalimumab, there remained an increased risk of multifocal tumor development and high-grade disease at diagnosis but not in patients exposed to infliximab or etanercept (Supplementary Table 4).

**Table 1:** Propensity score-adjusted risk of urologic cancers in TNF-I exposed patients

		Relative risk (RR)	95% CI	*p*-value
Prostate				
	Number of Elevated PSA’s	1.23	(1.08, 1.39)	0.001*
	Number of Prostate MRI’s	1.12	(0.96, 1.31)	0.157
	Number of PNB	1.86	(0.40, 8.72)	0.429
	Elevated PSA at Diagnosis	0.91	(0.81, 1.03)	0.120
	Clinical Stage	0.85	(0.67, 1.09)	0.195
	Biopsy Grade Group 1 vs. 2+	0.95	(0.84, 1.07)	0.392
	Biopsy Grade Group 2- vs. 3+	1.04	(0.86, 1.26)	0.691
	Prostatectomy TNM Stage	1.14	(0.84, 1.54)	0.413
	Prostatectomy Grade Group 1 vs. 2+	1.11	(1.01, 1.22)	0.036*
Bladder				
	Higher Pathologic Stage at Diagnosis	0.27	(0.11, 0.70)	0.007*
	Number of Tumors	1.98	(1.32, 2.97)	0.001*
	Grade at Diagnosis	1.39	(1.11, 1.72)	0.004*
	Radical Cystectomy	0.24	(0.09, 0.61)	0.003*
Kidney				
	Clinical Stage 2 or Greater	1.40	(0.91, 2.15)	0.130
	Clinical Stage 3 or Greater	2.01	(1.21, 3.33)	0.007*
	Pathologic Stage 2 or Greater	1.43	(0.95, 2.14)	0.087
	Pathologic Stage 3 or Greater	1.29	(0.85, 1.96)	0.231
	Partial Nephrectomy	1.09	(0.83, 1.44)	0.525
	Radical Nephrectomy	0.83	(0.63, 1.11)	0.208

*p < 0.05 considered statistically significant

## Discussion

Our data from 56,209 patients with chronic inflammatory conditions, 19,678 of whom were men, matched previous large registry studies [22]. Here, we identified 893 urologic malignancies in patients with chronic inflammatory conditions and confirmed our previous findings of an association between TNF-I exposed patients and lower PCa diagnosis risk [23], although those patients had higher risk of higher GG PCa. We also found an association between TNF-I exposed patients and increased risk of high-grade disease and multifocal tumor development for UCC and higher clinical stage disease in RCC. These analyses used TNF-I exposure as a time-dependent variable in an IPTW analysis to account for duration of TNF-I exposure, adding to the strength of these findings.

This association of decreased PCa diagnosis risk in TNF-I exposed patients agrees with Hellgren et al., who found a 50% PCa diagnosis risk reduction in TNF-I exposed patients in a nationwide Ankylosing Spondylitis dataset [9]. Other previous studies perform similarly superficial analyses, with many identifying a single index disease entity, not differentiating by drug exposure, grouping all malignancies together, or grouping all biologic therapies together as a single clinical entity [24–32]. Our paper builds on these findings by performing manual chart review on every patient with urologic malignancy across all chronic inflammatory conditions along with sub-analyses investigating tumor characteristics, adding significant context to the association between TNF-I exposure and PCa.

We found that despite overall lower PCa rates amongst TNF-I exposed patients, there was increased number of elevated PSA’s prior to diagnosis and an increased risk of higher GG at prostatectomy when exposed to a TNF-I. When isolating individual TNF-I drug exposure, this effect was largely driven by etanercept. The basic science literature surrounding TNF-I and prostate cancer suggests that (1) TNF is chronically produced at low levels within the prostate cancer microenvironment; (2) TNF levels in PCa correlate with extent of disease, resistance to therapy, and metastasis; and (3) TNF leads to increased CCR7 expression to promote lymph node metastasis [33–35]. These findings suggest a mechanism that could explain the associations found between TNF inhibition and decreased overall PCa rates. The association with higher grade PCa in these same patients could also from growth in the absence of TNF stimulation, although this study was not equipped to evaluate causality for this relationship. Alternatively, another possible explanation is that while chronic immune-mediated inflammation can increase PCa risk, decreased immune surveillance could lead to worsening of malignant disease. This is the premise of immunotherapy and provides a possible explanation for chronic TNF inhibition and its association with higher GG PCa [36]. This proposed cellular mechanism agrees with our data which should be further studied to elucidate the possible relationship between TNF-I exposure and PCa modulation.

For the association of higher stage RCC in TNF-I exposed patients, this aligns well with the current literature regarding the immunosensitivity of RCC. RCC is known to evade the immune system via eliciting immune-inhibitory cells into its microenvironment [37]. As such, the mainstay of treatment in advanced RCC is immunotherapy and many patients have significant improvements in their disease burden in response to these therapies [38]. Our findings of increased clinical stage of RCC in TNF-I exposed patients agrees with this body of evidence for the immunogenicity of RCC and suggests that a larger study with more power could prospectively confirm an increased risk of higher pathologic RCC stage in TNF-I exposed patients and its effect on patient outcomes.

Our findings in UCC in TNF-I exposed patients are mixed although largely align with the existing literature. We found that in TNF-I exposed patients, there was an increased risk of multifocal tumor development and higher-grade tumors which was offset by the decreased risk of development of muscle invasive bladder cancer (MIBC). This association suggests a more complex immune microenvironment governing the development and progression of bladder cancer where TNF-I could possibly be associated with higher risk pathologic features in non-muscle invasive bladder cancer (NMIBC) but without higher risk of MIBC progression. This complex immune interplay aligns with NMIBC treatment starting with Bacillus Calmette-Guerin (BCG), which initiates a robust immune reaction that recruits granulocytes, natural killer cells, and CD4/CD8 lymphocytes to eliminate the tumor [39]. Similarly, advanced MIBC is commonly treated with immunotherapy [40]. Thus, our findings of an association between TNF-I exposure and increased grade and multifocality of UCC on diagnosis add to the established body of evidence regarding UCC immunogenicity and provide a basis for further investigation of a possible mechanism for this finding in TNF-I immunosuppression along with oncologic outcomes.

There are some limitations of the present study. Surveillance bias cannot be fully excluded in patients on TNF-I who may have greater contact with the healthcare system but none of the urologic cancer diagnosis risks match the expected pattern of surveillance bias. We investigated that for PCa by comparing number of screening PSA’s drawn, which yielded no evidence of surveillance bias. On the other hand, UCC and RCC are not screen-detected cancers and, aside from Crohn’s disease, urinalysis and cross-sectional abdominal imaging are not standard surveillance strategies for chronic inflammatory diseases. We also attempted to mitigate this possibility by comparing total number of screening exams and year of RCC diagnosis between TNF-I exposed and unexposed cohorts and found no difference. Furthermore, while our analyses accounted for TNF-I exposure as a time-dependent variable, the medical records do not fully capture true TNF-I exposure time, as patients can choose to fill or not fill their prescriptions, receive medications outside of our health system, or have treatment interruptions. This could alter the TNF-I exposure timing in our calculations. Finally, for many chronic inflammatory disorders, typically more severe phenotypes progress to TNF-I therapy. It is possible that chronic inflammation induces cancer in the mild-moderate phenotypes and that TNF-I exposure lowers that risk closer to general population. This is a limitation, as we could not reliably stratify disease severity across multiple heterogenous index inflammatory diseases, however, previous studies have linked IBD to prostate cancer independent of severity.

## Conclusions

TNF-I exposure is associated with a decreased risk of PCa but higher risk of higher Gleason grade PCa. TNF-I exposure is not associated with increased risk of UCC or RCC diagnosis but was associated with increased risk of high-grade disease and multifocal tumor development for UCC and higher clinical stage disease in RCC. These findings suggests a need for prospective, multi-center studies to determine if TNF-I exposed patients have different risks of urologic malignancy.

## Supplementary Information

Below is the link to the electronic supplementary material.

Supplementary Material 1.

## Data Availability

Study protocol and statistical analysis plan are fully available in the text and supplement. After de-identification, individual participant data that underlie the study results and analytic code will be available immediately upon publication with no end date to researchers who provide a methodologically sound proposal. Proposals should be directed to [conor.driscoll@northwestern.edu] (mailto: conor.driscoll@northwestern.edu) . To gain access, data requestors must sign a data access agreement.
