# Supplementary material for: Urologic malignancy risk with chronic tumor necrosis factor-alpha inhibitor (TNF-I) exposure: a multicenter, retrospective cohort study
Source: World J Urol. 2026 Feb 26;44(1):202. doi: 10.1007/s00345-026-06309-0 (PMC12945975; doi:10.1007/s00345-026-06309-0)

Supplementary Materials

Supplementary Methods 1: Index Disease and Comorbidity ICD-9 and ICD-10 codes

*Chronic Inflammatory Conditions*

- Crohn’s Disease (CD): **ICD-9**=555.0-555.2, 555.9; **ICD-10**=K50.0, K50.1, K50.8, K50.9
- Ulcerative Colitis (UC): **ICD-9**=556.0-556.6, 556.8, 556.9; **ICD-10**=K51.0-K51.5, K51.8, K51.9
- Psoriasis (Ps)/Psoriatic Arthritis (PsA): **ICD-9**=696.0-696.5, 696.8; **ICD-10**=L40.0-L40.4, L40.8
- Rheumatoid Arthritis (RA): **ICD-9**=714.0-714.2, 714.30-714.33, 714.4; **ICD-10**=M05.0-M05.9
- Ankylosing Spondylitis (AS)/Spondyloarthritis (SpA): **ICD-9**=720.20-720.2, 720.8, 720.9; **ICD-10**=M45.0-M45.9
- Hidradenitis Suppurativa (HS): **ICD-9**=705.83; **ICD-10**=L73.2
- Uveitis (UV): **ICD-9**=364.00-364.05; **ICD-10**=H20.00-H20.05, H20.1

*Comorbidities*

- Hypertension (HTN): **ICD-9** = 401.0, 401.1, 401.9, 403.00, 403.01, 403.10, 403.11, 403.90, 403.91; **ICD-10** = I10, I11.0, I11.9, I12.0, I12.9
- Hyperlipidemia (HLD): **ICD-9** = 272.4; **ICD-10** = E78.0-E78.5
- Diabetes Mellitus: **ICD-9** = 250.0-250.9; **ICD-10** = E10 (Type 1, 84 sub-codes), E11 (Type II, 84 sub-codes)
- Congestive Heart Failure (CHF): **ICD-9** = 428.0, 428.1, 428.20-428.23, 428.30-428.33, 428.40-428.43, 428.9; **ICD-10** = I50.1-I50.4, I50.8, I50.9
- Coronary Artery Disease (CAD): **ICD-9** = 410.0-410.9 (all .00-.02 only), 411.0, 411.1, 411.81, 411.89, 412, 414.00-414.07, 414.10-414.12, 414.19,414.2-414.4, 414.8, 414.9; **ICD-10** = I20-I25
- Chronic Kidney Disease (CKD): **ICD-9** = 585.1-585.1, 585.9; **ICD-10** = N18.1-N18.6, N18.9
- Chronic Obstructive Pulmonary Disease (COPD): **ICD-9** = 491-494; **ICD-10** = J41-J44, J47
- HIV/AIDS: **ICD-9** = 042; **ICD-10** = B20
- *Lynch Syndrome: **ICD-9** = 758.5 (Other conditions due to autosomal abnormalities); **ICD-10** = Z15.02 (ovary), Z15.04 (endometrium), Z15.09 (genetic susceptibility to other malignant neoplasm)
- *Gardner Syndrome: **ICD-9** = 758.5 (Other conditions due to autosomal abnormalities); **ICD-10** = Z15.09(genetic susceptibility to other malignant neoplasm)
- *Li-Fraumeni Syndrome: **ICD-9** = 758.5 (Other conditions due to autosomal abnormalities); **ICD-10** = Z15.01 (breast), Z15.09 (genetic susceptibility to other malignant neoplasm)
- HPV: **ICD-9** = 078.10-078.12, 078.19, 079.4; **ICD-10** = B07.0, B07.8, B07.9
- EBV: **ICD-9** = 075; **ICD-10** = B27.90-B27.92, B27.99
- Hepatitis B (HBV): **ICD-9** = 070.20-070.23, 070.30-070.33; **ICD-10** = B16.0-B16.2, B16.9, B18.0, B18.1, B19.10, B19.11
- Hepatitis C (HCV): **ICD-9** = 070.41, 070.44, 070.51, 070.54, 070.70, 070.71; **ICD-10** = B17.10, B17.11, B18.2, B19.20, B19.21
- H. pylori: **ICD-9** = 041.86; **ICD-10** = B96.81

*comorbidity can only be identified by generic genetic abnormality ICD-9/10 code

Supplementary Methods 2: Urologic Malignancy ICD-9 and ICD-10 Codes.

*ICD-9 Codes:*

- Prostate: 185
- Testis: 186
- Bladder: 188
- Kidney: 189
- Corresponding in situ neoplasms: 230-234

*ICD-10 Codes:*

- Prostate: C61
- Testis: C62
- Kidney: C64-C65
- Bladder: C66-C67
- Corresponding in situ neoplasms: D00-D09

Supplementary Methods 3: Prostate Cancer Screening Variables

- Number of encounters for prostate cancer screening: ICD-10 Z12.5
- Initial PSA value
- Number of PSA tests: CPT 84152
- Number of transrectal diagnostic ultrasounds: CPT 76872/76942
- Number of prostate MRI: CPT 72197
- Number of prostate biopsies: CPT 55700/55706
- Abnormal digital rectal examination: ICD-9 796.4
- Prostate induration: ICD-9 600.10/600.11

Supplementary Table 1: System Wide Demographics of Diagnosed Prostate Cancer Patients

|  | TNF-I Unexposed (n=490) | TNF-I Exposed (n=53) | P-value |
| --- | --- | --- | --- |
| Age | 61 (54, 69) | 61 (55, 67) | >0.9 |
| Follow up time (months) |  |  |  |
| Overall | 87 (38, 144) | 101 (56, 168) | <0.001 |
| Post-exposure | - | 46 (21, 78) |  |
| Race |  |  | 0.8 |
| Black | 58 (12%) | 7 (13%) |  |
| White | 421 (86%) | 46 (87%) |  |
| Other | 11 (2.2%) | 0 (0%) |  |
| Smoking |  |  | 0.059 |
| Current | 40 (8.2%) | 2 (3.8%) |  |
| Former | 214 (44%) | 16 (30%) |  |
| Never | 236 (48%) | 35 (66%) |  |
| TNF-I |  |  | <0.001 |
| Adalimumab | 0 (0%) | 26 (49%) |  |
| Infliximab | 0 (0%) | 10 (19%) |  |
| Etanercept | 0 (0%) | 14 (26%) |  |
| Certolizumab | 0 (0%) | 2 (3.8%) |  |
| Golimumab | 0 (0%) | 1 (1.9%) |  |
| PSA at diagnosis |  |  |  |
| Median (IQR) | 5.98 (4.26, 8.60) | 5.00 (3.80, 7.93) | 0.2 |
| <4 | 69 (14%) | 12 (23%) | 0.3 |
| 4-10 | 225 (46%) | 25 (47%) |  |
| >10 | 69 (14%) | 6 (11%) |  |
| Unknown | 127 (26%) | 10 (19%) |  |
| Grade Group on Biopsy |  |  | 0.6 |
| 1 | 165 (34%) | 22 (42%) |  |
| 2 | 100 (20%) | 10 (19%) |  |
| 3 | 70 (14%) | 5 (9%) |  |
| 4 | 33 (7%) | 4 (8%) |  |
| 5 | 29 (6%) | 5 (9%) |  |
| Unknown | 93 (19%) | 7 (13%) |  |
| # of positive cores |  |  | 0.5 |
| 1 | 76 (16%) | 14 (26%) |  |
| 2-6 | 194 (40%) | 20 (38%) |  |
| 7-10 | 37 (8%) | 5 (9%) |  |
| >10 | 20 (4%) | 2 (4%) |  |
| Unknown | 163 (32%) | 12 (23%) |  |
| Adverse Pathology |  |  |  |
| EPE | 48 (10%) | 12 (23%) | 0.4 |
| SVI | 15 (3%) | 2 (4%) | >0.9 |
| LVI | 10 (2%) | 4 (8%) | 0.3 |
| N1 | 7 (1%) | 3 (6%) | 0.4 |
| M1 | 37 (8%) | 4 (8%) | >0.9 |

*p < 0.05 considered statistically significant

Supplementary Table 2: System Wide Demographics of Diagnosed Renal Cell Carcinoma Patients

|  | TNF-I Unexposed (n=142) | TNF-I Exposed (n=30) | P-value |
| --- | --- | --- | --- |
| Age | 56 (49, 65) | 58 (50, 64) | 0.8 |
| Gender |  |  | 0.6 |
| Female | 74 (52%) | 14 (47%) |  |
| Male | 68 (48%) | 16 (53%) |  |
| Follow up (months) |  |  |  |
| Overall | 87 (38, 144) | 101 (56, 168) | <0.001* |
| Post-exposure | - | 46 (21, 78) |  |
| Race |  |  | 0.7 |
| Black | 23 (16%) | 5 (17%) |  |
| White | 117 (82%) | 24 (80%) |  |
| Other | 2 (2%) | 1 (3%) |  |
| Smoking Status |  |  | 0.3 |
| Current | 21 (15%) | 7 (23%) |  |
| Former | 55 (39%) | 8 (27%) |  |
| Never | 66 (46%) | 15 (50%) |  |
| Clinical Stage at Dx |  |  | 0.5 |
| T1 | 96 (68%) | 22 (73%) |  |
| T2 | 7 (5%) | 0 (0%) |  |
| T3 | 7 (5%) | 3 (10%) |  |
| T4 | 4 (3%) | 0 (0%) |  |
| N1/M1 | 5 (4%) | 0 (0%) |  |
| Unknown | 23 (24%) | 5 (17%) |  |
| Surgical Approach |  |  | 0.065 |
| PNx | 59 (42%) | 17 (57%) |  |
| RNX | 63 (44%) | 8 (26%) |  |
| None | 20 (14%) | 5 (17%) |  |
| Histologic Subtype |  |  | 0.3 |
| Chromophobe | 5 (4%) | 2 (7%) |  |
| Clear cell | 92 (65%) | 22 (73%) |  |
| Papillary | 18 (13%) | 1 (3%) |  |
| Variant | 7 (5%) | 0 (0%) |  |
| Unknown | 20 (14%) | 5 (17%) |  |
| Recurrence | 14 (10%) | 1 (3%) | 0.5 |
| Mortality | 23 (24%) | 0 (0%) | 0.015* |
| Average Year of Diagnosis | 2012 | 2014 |  |
| Number of Screening Colonoscopies | 1 (0-2) | 0 (0-2) | 0.8 |
| Number of Screening Low-dose CT scans | 0 (0-1) | 0 (0-3) | 0.092 |
| Total Screening Tests (Colonoscopies and Low-dose CT scans) | 2 (1-3) | 2 (1-6) | 0.4 |

*p < 0.05 considered statistically significant

Supplementary Table 3: System Wide Demographics of Diagnosed Urothelial Cell Carcinoma Patients

|  | TNF-I Unexposed (n=162) | TNF-I Exposed (n=16) | P-value |
| --- | --- | --- | --- |
| Age | 65 (57, 73) | 65 (56, 68) | 0.6 |
| Gender |  |  | 0.2 |
| Female | 59 (36%) | 3 (19%) |  |
| Male | 103 (64%) | 13 (81%) |  |
| Follow up time (months) |  |  |  |
| Overall | 87 (38, 144) | 101 (56, 168) | <0.001* |
| Post-exposure | - | 46 (21, 78) |  |
| Race |  |  | >0.9 |
| Black | 13 (8%) | 1 (6%) |  |
| White | 142 (88%) | 15 (94%) |  |
| Other | 7 (4%) | 0 (0%) |  |
| Smoking Status |  |  | 0.7 |
| Current | 23 (14%) | 1 (6%) |  |
| Former | 83 (51%) | 8 (50%) |  |
| Never | 56 (35%) | 7 (44%) |  |
| Location |  |  | 0.2 |
| Lower Tract | 127 (78%) | 15 (94%) |  |
| Upper Tract | 35 (22%) | 1 (6%) |  |
| Clinical Stage at Dx |  |  | 0.4 |
| ≤T1 | 108 (67%) | 14 (87%) |  |
| ≥T2 | 35 (21%) | 2 (13%) |  |
| Unknown | 19 (12%) | 0 (0%) |  |
| Grade at Dx |  |  | >0.9 |
| Low | 57 (35%) | 6 (38%) |  |
| High | 84 (52%) | 9 (56%) |  |
| Unknown | 21 (13%) | 1 (6%) |  |
| Number of Tumors |  |  | 0.4 |
| 1 | 94 (58%) | 10 (62%) |  |
| 2+ | 35 (22%) | 6 (38%) |  |
| Unknown | 33 (20%) | 0 (0%) |  |
| Histologic Subtype |  |  | >0.9 |
| Urothelial | 140 (87%) | 15 (94%) |  |
| Variant | 10 (6%) | 1 (6%) |  |
| Unknown | 12 (7%) | 0 |  |
| LN+ | 17 (10%) | 0 (0%) | 0.063 |
| Metastasis | 20 (12%) | 0 (0%) | 0.2 |
| Mortality | 45 (28%) | 2 (13%) | 0.2 |
| Number of Screening Colonoscopies | 1 (0-2) | 1 (0-2.5) | >0.9 |
| Number of Screening Low-dose CT scans | 0 (0-2) | 0.5 (0-4.5) | 0.6 |
| Total Screening Tests (Colonoscopies and Low-dose CT scans) | 2 (1-4) | 4 (1-4.5) | 0.3 |

*p < 0.05 considered statistically significant

Supplementary Table 4: Propensity Score-Adjusted Risk of Urologic Cancers for Adalimumab

|  |  | Relative Risk (RR) | 95% CI | p-value |
| --- | --- | --- | --- | --- |
| Prostate |  |  |  |  |
|  | Number of Elevated PSA’s | 0.90 | (0.77, 1.05) | 0.163 |
|  | Number of Prostate MRI’s | 0.65 | (0.53, 0.80) | <0.001* |
|  | Number of PNB | 1.73 | (0.08, 35.87) | 0.723 |
|  | Elevated PSA at Diagnosis | 0.96 | (0.85, 1.08) | 0.516 |
|  | Clinical Stage | 0.45 | (0.33, 0.61) | <0.001* |
|  | Biopsy Grade Group 1 vs. 2+ | 1.16 | (1.04, 1.29) | 0.008* |
|  | Biopsy Grade Group 2- vs. 3+ | 0.67 | (0.53, 0.85) | 0.001* |
|  | Prostatectomy TNM Stage | 0.47 | (0.29, 0.76) | 0.002* |
|  | Prostatectomy Grade Group 1 vs. 2+ | 0.93 | (0.80, 1.07) | 0.286 |
| Bladder |  |  |  |  |
|  | Number of Locations 1 vs. 2+ | 1.79 | (1.16, 2.77) | 0.009* |
|  | Grade at Diagnosis | 1.49 | (1.20, 1.84) | <0.001* |
|  | Radical Cystectomy | <0.01 | (0, Inf) | 0.989 |
|  |  |  |  |  |
| Kidney |  |  |  |  |
|  | Clinical Stage 2 or Greater | 0.99 | (0.63, 1.57) | 0.970 |
|  | Clinical Stage 3 or Greater | 1.42 | (0.84, 2.42) | 0.191 |
|  | Pathologic Stage 2 or Greater | 0.88 | (0.56, 1.38) | 0.568 |
|  | Pathologic Stage 3 or Greater | 0.88 | (0.56, 1.38) | 0.568 |
|  | Partial Nephrectomy | 1.45 | (1.14, 1.85) | 0.002* |
|  | Radical Nephrectomy | 0.40 | (0.28, 0.59) | <0.001* |

*p < 0.05 considered statistically significant

Supplementary Table 5: Propensity Score-Adjusted Risk of Urologic Cancers for Infliximab

|  |  | Relative Risk (RR) | 95% CI | p-value |
| --- | --- | --- | --- | --- |
| Prostate |  |  |  |  |
|  | Number of Elevated PSA’s | 1.80 | (1.58, 2.06) | <0.001* |
|  | Number of Prostate MRI’s | 1.83 | (1.60, 2.09) | <0.001* |
|  | Elevated PSA at Diagnosis | 0.76 | (0.66, 0.87) | <0.001* |
|  | Clinical Stage | 2.22 | (1.84, 2.68) | <0.001* |
|  | Biopsy Grade Group 1 vs. 2+ | 0.70 | (0.61, 0.81) | <0.001* |
|  | Prostatectomy TNM Stage | 2.18 | (1.69, 2.82) | <0.001* |
|  | Prostatectomy Grade Group 1 vs. 2+ | 0.63 | (0.55, 0.72) | <0.001* |
| Bladder |  |  |  |  |
|  | Higher Pathologic Stage at Diagnosis | 0.85 | (0.44, 1.66) | 0.639 |
|  | Radical Cystectomy | 0.79 | (0.41, 1.50) | 0.468 |
| Kidney |  |  |  |  |
|  | Clinical Stage 2 or Greater | 1.31 | (0.75, 2.29) | 0.335 |
|  | Clinical Stage 3 or Greater | 1.87 | (1.01, 3.48) | 0.047* |
|  | Pathologic Stage 2 or Greater | 1.16 | (0.67, 2.01) | 0.588 |
|  | Pathologic Stage 3 or Greater | 1.16 | (0.67, 2.01) | 0.588 |

*p < 0.05 considered statistically significant

Supplementary Table 6: Propensity Score-Adjusted Risk of Urologic Cancers for Etanercept

|  |  | Relative Risk (RR) | 95% CI | p-value |
| --- | --- | --- | --- | --- |
| Prostate |  |  |  |  |
|  | Number of Elevated PSA’s | 1.08 | (0.94, 1.24) | 0.278 |
|  | Number of Prostate MRI’s | 0.69 | (0.57, 0.85) | <0.001* |
|  | Elevated PSA at Diagnosis | 0.52 | (0.43, 0.62) | <0.001* |
|  | Clinical Stage | 0.47 | (0.34, 0.66) | <0.001* |
|  | Biopsy Grade Group 1 vs. 2+ | 0.88 | (0.77, 0.99) | 0.046* |
|  | Biopsy Grade Group 2- vs. 3+ | 1.53 | (1.29, 1.82) | <0.001* |
|  | Prostatectomy Grade Group 1 vs. 2+ | 1.18 | (1.07, 1.29) | <0.001* |
| Bladder |  |  |  |  |
|  | Number of Tumors | <0.01 | (0, Inf) | 0.991 |
|  | Grade at Diagnosis | 1.21 | (0.97, 1.52) | 0.090 |
|  | Radical Cystectomy | <0.01 | (0, Inf) | 0.991 |
| Kidney |  |  |  |  |
|  | Pathologic Stage 2 or Greater | 0.90 | (0.24, 3.42) | 0.881 |
|  | Radical Nephrectomy | 0.25 | (0.06, 1.03) | 0.054 |

*p < 0.05 considered statistically significant

Supplementary Figure 1: Forest Plot of Odds Ratio of Prostate Cancer for Each Autoimmune Disease Type


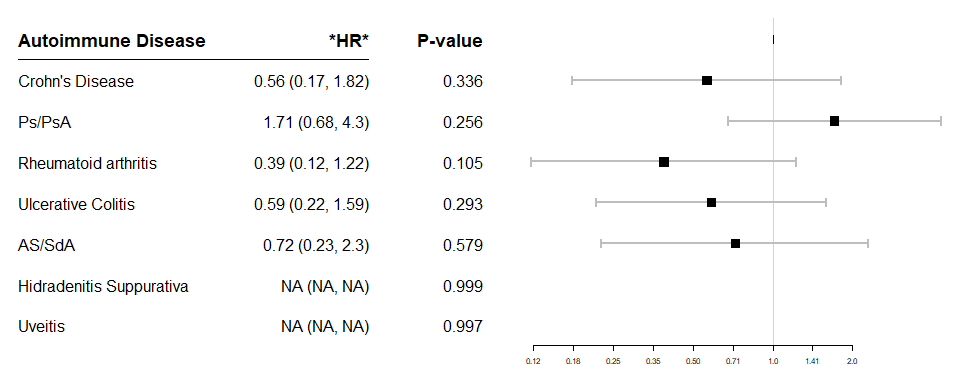


Supplementary Figure 2: Multivariable regression of PCa diagnosis risk controlled for number of screening PSA’s (Sensitivity Analysis)


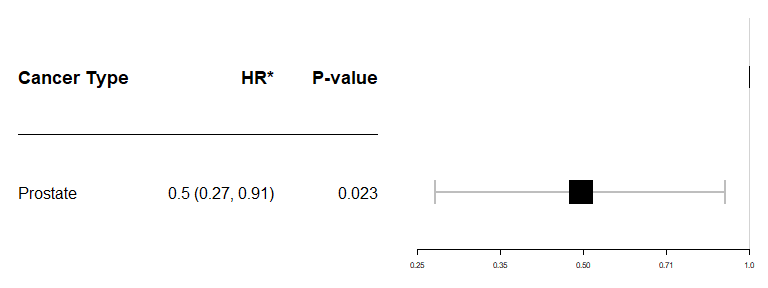

Supplement: Supplementary file 1 — Supplementary Material 1. [file 345_2026_6309_MOESM1_ESM.docx]
